# Promoting health literacy through the teach back method among Iranian health ambassadors: A randomized controlled trial*

**DOI:** 10.17533/udea.iee.v40n1e17

**Published:** 2022-03-31

**Authors:** Ahmad Sotoudeh, Mojtaba Fattahi Ardakani, Mohammad Saeed Jadgal, Ali Asadian, Morad Ali Zareipour

**Affiliations:** 1 Department of Public Health, Bushehr University of Medical Sciences, Bushehr, Iran. Email: Sotoudeh_ahmad@yahoo.com Department of Public Health Bushehr University of Medical Sciences Bushehr Iran Sotoudeh_ahmad@yahoo.com; 2 Diabetes Center Research, Shahid Sadoughi University of Medical Sciences, Yazd, Iran. Email: mjfattahi57@gmail.com Diabetes Center Research Shahid Sadoughi University of Medical Sciences Yazd Iran mjfattahi57@gmail.com; 3 Department of Public Health, Iranshahr University of Medical Sciences, Chabahar, Iran. Email: jadgal15@gmail.com Department of Public Health Iranshahr University of Medical Sciences Chabahar Iran jadgal15@gmail.com; 4 Social Determinants in Health Promotion Research Center, Hormozgan University of Medical Sciences, Bandar Abbas, Iran. Email: a.assadian.a@gmail.com Social Determinants in Health Promotion Research Center Hormozgan University of Medical Sciences Bandar Abbas Iran a.assadian.a@gmail.com; 5 Department of Public Health, School of Health, Khoy University of Medical Sciences, Khoy, Iran. Email: z.morad@yahoo.com. Department of Public Health School of Health Khoy University of Medical Sciences Khoy Iran z.morad@yahoo.com

**Keywords:** health literacy, teach-back communication, access to information, health behavior., alfabetización en salud, método teach-back, acceso a la información, conductas relacionadas con la salud., letramento em saúde, comunicação para apreensão de informação, acesso à informação, comportamentos relacionados com a saúde.

## Abstract

**Objective.:**

Describe the effect the teach back method on promoting the health literacy of health ambassadors in Urmia County in 2020.

**Methods.:**

In the present quasi-experiment, 200 persons over 14 years old participated. They were divided into two research groups, a control (*n*=100) and an intervention (*n*=100). The sampling method was simple randomization and the data collection instrument was a questionnaire comprised of demographic information and health literacy (HELIA). The educational intervention took 4 sessions each 45 minutes in length following the teach back method. The questionnaire-based data were collected once before the intervention and once again three months after the intervention.

**Results.:**

The present findings showed that 54% of the control group and 50% of the intervention group had a good or very good level of health literacy before the intervention(*p*>0.05). However, after the intervention, 52% of the control and 78% of the intervention group had a good or very good level of health literacy. The present findings revealed that the mean scores of health literacy dimensions (access to information, reading, understanding, appraisal, decision-making) and the overall health literacy score were significantly higher in the intervention group than the control (after the intervention). Wilcoxon’s test results showed that the mean difference of the overall health literacy scores and the dimensions before and after the intervention were statistically significant (*p*<0.001).

**Conclusion.:**

In the light of the present findings, we can conclude that participatory methods and the teach back method can improve health literacy, acquire reliable information and adopt healthy behaviors.

## Resumen

**Objetivo.:**

Describir el efecto del método teach-back en la promoción de la alfabetización en salud de los embajadores de la salud en el condado de Urmia, Irán en 2020**.**

**Métodos.:**

En el presente estudio cuasiexperimental participaron 200 personas mayores de 14 años. Mediante muestreo de aleatorización simple se asignaron los grupos de investigación: de control (*n*=100) y de intervención (*n*=100). Se empleó un instrumento para la recogida de datos que contenía información demográfica y el instrumento *Health Literacy for Iranian Adults* -HELIA-. La intervención educativa consistió en 4 sesiones de 45 minutos, siguiendo el método teach- back. Los datos basados en el cuestionario se recogieron antes de la intervención y tres meses después de la misma.

**Resultados.:**

Los hallazgos mostraron que el 54% del grupo de control y el 50% del grupo de intervención tenían un nivel bueno o muy bueno de conocimientos sobre alfabetización en salud antes de la intervención (*p*>0.05). Sin embargo, después de la intervención, el 52% del grupo de control y el 78% del grupo de intervención tenían un nivel bueno o muy bueno de conocimientos sanitarios. Los presentes resultados revelaron que las puntuaciones medias de las dimensiones de la alfabetización en salud *acceso a la información, lectura, comprensión, valoración, toma de decisiones* y de la puntuación global fueron significativamente mayores en el grupo de intervención que en el de control a los 3 meses después de la intervención. Finalmente, los resultados de la prueba de Wilcoxon mostraron que la diferencia media de las puntuaciones de la alfabetización en salud y de las dimensiones antes y después de la intervención eran estadísticamente significativas (*p*<0.001).

**Conclusión.:**

A la luz de los resultados, se pudo concluir que la aplicación del método teach-back puede mejorar los conocimientos sobre la alfabetización en salud, además de ayudar a adquirir información fiable y a adoptar comportamientos saludables.

## Resumo

**Objetivo.:**

Descrever o efeito do método teach-back na promoção da alfabetização em saúde dos embaixadores da saúde no condado de Urmia, Irã em 2020**.**

**Métodos.:**

No presente estudo quase-experimental participaram 200 pessoas maiores de 14 anos. Mediante amostragem de aleatória simples foram designados aos grupos de investigação: de controle (*n*=100) e de intervenção (*n*=100). Se empregou um instrumento para o recolhimento de dados que continha informação demográfica e o instrumento Health Literacy for Iranian Adults -HELIA-. A intervenção educativa consistiu em 4 sessões de 45 minutos, seguindo o método teach- back. Os dados baseados no questionário se recolheram antes da intervenção e três meses depois dela.

**Resultados.:**

As descobertas mostraram que 54% do grupo de controle e 50% do grupo de intervenção tinham um nível bom ou muito bom de conhecimentos sobre alfabetização em saúde antes da intervenção (*p*>0.05). Porém, depois da intervenção, 52% do grupo de controle e 78% do grupo de intervenção tinham um nível bom ou muito bom de conhecimentos sanitários. Os presentes resultados revelaram que as pontuações médias das dimensões da alfabetização em saúde *acesso à informação, leitura, compreensão, valoração, toma de decisões* e da pontuação global foram significativamente maiores no grupo de intervenção que no de controle aos 3 meses depois da intervenção. Finalmente, os resultados da prova de Wilcoxon mostraram que a diferença média das pontuações da alfabetização em saúde e das dimensões antes e depois da intervenção eram estatisticamente significativas (*p*<0.001).

**Conclusão.:**

À luz dos resultados, se pôde concluir que a aplicação do método teach-back pode melhorar os conhecimentos sobre a alfabetização em saúde, além de ajudar a adquirir informação confiável e a adotar comportamentos saudáveis.

## Introduction

Health literacy is a major moderator in the effectiveness of health education interventions.(1) Health education and healthliteracy are correlated. In other words, health literacy is a main consequence of health education interventions, but implementing health literacy interventions regardless of the perceived health literacy adversely affects the effectiveness and benefits of these interventions.(2) Health literacy refers to the ability to obtain, process and understand basic health information and services to decide appropriately on health issues. It includes the skills of reading, hearing and analyzing health information, making appropriate health decisions and being able to use these skills in health-related conditions.(3,4) Moreover, low health literacy is a barrier to participation in health education programs.(3) A body of related literature showed that a good level of health literacy can increase the effectiveness of health promotion programs and can improve individuals’ health.(3) Educating people in society is a time-consuming task in health services. It is recommended to explore the effectiveness of different educational methods in this regard.(5) Effective education requires the use of educational methods whose effectiveness is already proven. Among them is the evidence-based teach back method, a comprehensive multi-dimensional method used to obtain and retain information. It is also considered an interactive educational method in which the learner should develop the skills of obtaining and using the instructed information. This method also reduces the risk of misunderstanding the essential information an individual needs in risky conditions.(6)

This method takes into account participation, cooperation, social acceptance and recall. The learner is expected to develop the required skills of teaching what s/he has already learned. The distinctive feature of this method is that the effectiveness of what the learner manages to teach shows his/her efficiency and the effectiveness of instructions.(7) Another benefit of this method is self-appraisal. That is to say that before anyone else judges the quality of instructions, the learner him/herself does so. Therefore, this method has a corrective and re-educational feature.(8) In their research, Oshvandi et al. reported the effectiveness of the teach back method in promoting self-care behaviors in patients with diabetes type 2.(9)

Promoting health literacy is the greatest hope for decreasing the load of diseases and mortalities worldwide. Promoting health literacy can improve self-care and life quality, optimal use of health services and, thus, reduce health costs.(2) Therefore, since the health ambassador comprehensive plan was implemented in urban and rural healthcare centers, health ambassadors have been considered as the main educators in families. Health ambassadors are volunteers selected from households receiving services from healthcare centers. They might be students or working members of society who acquire health-related knowledge and transfer it to other family members and the community to which they belong.(10) Empowering family health ambassadors as elected representatives will be effective in promoting health literacy through the teach back method. Because with this training method, health ambassadors will learn health literacy materials in their own language and will easily transmit these learned materials in their own language to family members and thus will promote family health. Considering the distinctive features of the teach back method, the present research aimed to explore the effect of this method on promoting health ambassadors’ health literacy in Urmia County in 2020.

## Methods

The present clinical trial used randomization in both research groups. It was quasi-experimental in type and was conducted in Urmia County in Iran in 2020. The sample size was determined in the light of the existing literature(11) using G-Power, an effect size of 40%, a standard deviation of 30.5±2.8 and 19.27±3.3 for the first and second groups respectively, a confidence interval of 95% , a test power of 80% and a 10% attrition rate for each group. The final sample size was estimated at 100. For the sampling, firstly, Urmia County was divided into the north and the south parts. Among the healthcare centers in each part, 10 were randomly selected and were further randomly divided into two groups (*n*=5) as the control and intervention. In the next step, among the list of health ambassadors’ names, 20 were selected from each center through simple randomization.

Being a health ambassador according to the national protocol (a person from each household who has at least Sixth elementary), having a health ambassador file in the center and health center, satisfaction with attending the study were the criteria for inclusion in the study.

The data were collected using a questionnaire in two parts. The first part enquired about the respondents’ demographic information (age, education, occupation, …) and the second part was HELIA, exploring the health literacy along five dimensions (reading, access to information, understanding, appraisal, health decisions/behavior) with 33 items. The raw scores of the 5 dimensions were then turned into a standard score ranging from 0 to 100. A score of 0-50 was interpreted as very poor, 50.1-66 as poor, 66.1-84 as good and 84.1-100 as very good health literacy. The psychometrics of this questionnaire were measured by Montazeri *et al.* and the validity was substantiated.(12) The validity of the HELIA questionnaire was confirmed using content validity. Reliability was also confirmed by calculating Cronbach's alpha coefficient (α=88).

Those who met the inclusion criteria entered the study. In order to prevent knowledge transfer between the two research groups, the health ambassadors of 5 healthcare centers were assigned to the control and 5 others to the intervention group. They were then contacted through phone calls to participate in the research. The purpose of study was revealed and an informed letter of consent was signed. The participants were asked to come again on certain dates as planned. At first, the pretest questionnaire was completed through an interview by the researcher and then the educational program was run in 4 sessions (each 45 minutes long) to teach health literacy (25 minutes of education, 20 minutes of teach back). The educational content of each session was taught face to face to health ambassadors through the teach back method along with a training using reliable sources. The ambassadors were then asked to recite the educational content in their own language. If the content showed not to be correctly understood by the health ambassador, the content was taught again. (Table 1).

Before the educational intervention, the data were collected from both research groups via the questionnaires and then the educational intervention was performed for the intervention group by TB method and for the control group, routine education was performed and Questionnaires for both groups were completed again after 3 months of education. To analyze the data in SPSS19 and compare the mean scores of the dimensions of health literacy in the pre- and post-tests in each group, paired-samples *T*-test was run. For between-group comparison, independent-samples *T*-test was run. The inclusion criteria were: being a health ambassador based on the national protocol (a literate household member with at least a junior high school degree), having a health ambassador’s record in the healthcare center and willingness to participate in the research.

**Table 1:** Summary of Educational Content of Health Literacy Intervention Group Sessions in Health Ambassadors by Teach-Back Method

Sessions	Educational content
First Session	**Educational topic title: *Reading***
	Familiarity of ambassadors with the objectives of the research, familiarity with reading educational materials on health, reading written instructions of doctors, dentists and health workers about diseases, reading medical and dental forms (such as patient admission form, consent form, file formation, etc. in hospitals and Medical centers), reading the instructions in the handbook and preparation before the examination, ultrasound or radiology.
Second Session	**Educational topic title: *Access to information***
	Familiarity with the principles of access to information on healthy eating, mental health, chronic diseases, infectious diseases, harms and dangers of smoking.
Third Session	**Educational topic title: *Understanding, appraisal***
	Familiar with the meaning and concept: Materials written in medical and dental forms, signs and materials written on billboards in hospitals and clinics, how to use drugs and instructions for taking drugs, contents written in the guide sheet before the test, ultrasound or radiology.
Fourth Session	**Educational topic title: *Health decisions/behavior***
	- Familiarity with how to properly assess the information provided related to health on the Internet, television and radio, friends and relatives.
	- Avoid doing things or consuming substances that endanger your health.
	- Perform annual check-ups (periodic examinations) to assess health
	- Use a seat belt while driving
	- Take care of your health in any job and situation

## Results

In the present research, 200 health ambassadors participated, half in the intervention group (*n*=100) and half in the control (*n*=100). The mean and standard deviation of the participants’ age in the intervention and control groups were 37.35±9.57 and 38.45±9.78 years, respectively. No statistically significant difference was found between the control and intervention groups in terms of age, sex, education, marital status and occupation. Thus, the two groups were homogeneous (Table 2).

**Table 2:** Comparison of demographic variables between the intervention and control groups

Variable		**Intervention group *n* (%)**	**Control group *n* (%)**	***p*-value**
Age	15-25	9 (9)	8 (8)	0.37
	25-35	37 (37)	39 (39)	0.37
	35-45	33 (33)	33 (33)	0.37
	46 +	21 (21)	20 (20)	0.37
Sex	Male	24 (24)	25 (25)	0.17
	Female	76 (76)	75 (75)	0.17
Marital status	Single	12 (12)	13 (13)	0.08
	Married	86 (86)	84 (84)	0.08
	Divorced/widowed	2 (2)	3 (3)	0.08
Occupation	Housewife	42 (42)	44 (44)	0.13
	Clerk	54 (54)	51 (51)	0.13
	Freelance	4 (4)	5 (5)	0.13
Education	Junior high school	42 (42)	39 (39)	0.09
	Diploma	39 (39)	43 (43)	0.09
	Academic degree	19 (19)	18 (18)	0.09

The present findings showed that 54% of the control group and 50% of the intervention group enjoyed a good or very good level of health literacy in the pretest. However, in the posttest, 52% of the control and 78% of the intervention group had good or very good health literacy (Table 3).

**Table 3:** Comparison of health literacy between the intervention and control groups in the pre- and post-test

Variable	Level	Control *n* (%)	Intervention *n* (%)
Health literacy in pre-test	Very poor	27 (27)	23 (23)
	poor	23 (23)	25 (25)
	Good	28 (28)	27 (27)
	Very good	22 (22)	25 (25)
Health literacy in the post-test	Very poor	22 (22)	7 (7)
	Poor	24 (24)	15 (15)
	Good	30 (30)	29 (29)
	Very good	24 (24)	49 (49)

The present findings showed that the mean scores of all dimensions of health literacy (access to information, reading, understanding, appraisal, decision making) and the total health literacy score were increased in the intervention group in the post-test. Wilcoxon’s test results showed that the mean difference of the total health literacy score and the dimensions between the pre- and post-test were statistically significant (*p*<.05). The mean health literacy score was increased after the intervention. However, no statistically significant difference was found within the control group in the pre- and post-test (p>.05). Mann-Whitney *U*-test revealed no statistically significant difference between the control and intervention group in the pre-test. Yet, the between-group difference in the post-test was statistically significant for the overall health literacy and the dimensions of health literacy (Table 4).

**Table 4:** Comparison of the mean health literacy scores in the two research groups in the pre- and post-test

Variable		Pre-test	Post-test	
Health literacy dimension	Group	mean±SD	mean±SD	p-value
Access to information	Intervention	12.82±3.80	13.63±4.89	<0.001
	Control	12.54±3.14	12.34±4.11	0.81
	*p*-value	0.57	< 0.001	
Reading	Intervention	10.62±3.39	14.82±3.37	<0.001
	Control	11.52±3.52	12.02±3.58	0.49
	*p*-value	0.83	<0.001	
Understanding	Intervention	11.75±4.31	13.86±5.91	<0.001
	Control	11.01±3.89	12.04±3.22	0.087
	*p*-value	.37	<.001	
Appraisal	Intervention	15.48±3.48	19.18±3.53	<0.001
	Control	15.1±3.51	15.84±3.51	0.2
	*p*-value	0.14	0.001	
Decision-making	Intervention	21.39±7.05	23.56±8.83	<0.001
	Control	21.78±6.05	22.02±6.05	0.06
	*p*-value	0.13	<0.001	
Total health literacy	Intervention	58.97±18.52	69.13±22.74	<0.001
	Control	57.77±17.54	58.76±18.41	0.1
	*p*-value	0.3	<0.001	

## Discussion

The present research aimed to explore the effect of the teach back method on health ambassadors’ health literacy in Urmia County. The findings revealed that educating through the teach back method can improve health literacy and promote preventive behaviors, an evidence for the effectiveness of the educational content in promoting health literacy. The mean health literacy score was significantly increased in the intervention group in the post-test. The difference between the mean health literacy score of the two groups was also statistically significant after the intervention. These findings are consistent with a body of research by Tal et al., Mirzaei et al. and Eftekhari *et al.*(14-15) these researchers showed that education can increase the level of health literacy, and that poor health literacy can be promoted through health education. As for the potential factors accounting for the rise of health literacy, it can be concluded that health literacy, similar to awareness, is a cognitive construct and poor health literacy is a definite concept of a low awareness of how to access information and then appraise it and decide on it to show the healthy behavior. In many educational methods, information is provided to different populations superficially with minor effects on the audience’s knowledge. Limited knowledge cannot empower an individual systematically. Low power impedes the occurrence of any controversial issue to the individual. Eventually, s/he cannot tell apart what is right and wrong.(16)

The present research also showed an increase in the number of participants with a good or very good level of health literacy in the intervention group in the post-test, which proves the effectiveness of the educational intervention. Thus, an educational intervention appropriate for the participants can increase the level of health literacy and the teach back method can be considered an effective and appropriate method.(17) The present findings also showed a statistically significant increase in the level of health literacy in the intervention group after the teach back method. Therefore, a greater awareness and knowledge of the principles of healthy lifestyle can enable people to know their environment and the existing health resources better. Promoting health literacy can be considered a main predictor of an improved health condition.(18,19) Moreover, after the educational intervention, there was a statistically significant increase in the mean understanding score of the intervention group compared to the control. Similarly, Smith et al. reported a more positive appraisal of the information resources than the control, and also a better understanding of the health information. (20) Kahtari et al. reported a higher mean score of understanding (a dimension of health literacy among female students after the educational intervention).(18)

The present research showed that the educational intervention managed to increase the mean scores of the dimensions of health literacy and decision-making. Many members of the society are faced with different types of health-related information some of which are neither accurate nor reliable.(21) Using unreliable information is considered a risk factor. Yet many people resort to these unreliable information sources and put their life and health at risk.(22) As defined by Nutbeam,(23) the critical health literacy concept is associated with a high level of recognition and social skill and is necessary for data analysis. Therefore, increasing the level of health literacy especially in appraisal and decision-making can affect individuals’ correct or incorrect analysis of information and appraisal of the quality and reliability of information. A good level of critical health literacy can be effective in the qualitative appraisal of health information especially weblogs and social media.(24,25) Furthermore, an increase in health literacy can be followed by a higher self-efficacy in different dimensions of health literacy and health consequences such as life quality.(26,27)

In the light of the present findings, we can also conclude that health literacy is a combination of a wide range of skills, capabilities and competencies in acquiring medical or health-related information, reading this information, understanding or interpreting it and sometimes making decisions on or applying this information. This is how health literacy can affect the adoption of preventive behaviors. In other words, through increasing the understanding and appraisal of the benefits of diagnostic and preventive behaviors, health literacy can be an effective factor involved in adopting these behaviors. Considering the positive effect of teach back method education method and considering the increasing growth of chronic diseases and the special role of nurses in educating and promoting community health literacy, it seems necessary to pay attention to this educational method. Nurses can be very effective in reducing adverse outcomes and promoting health by using this method. The use of this educational method as part of nurses' activities is recommended to reduce the problems of people in the community, especially health ambassadors.

One of the limitations of this study was the possibility of obtaining information while conducting research from other information sources (family members and media) other than the researcher's training in both control and intervention groups, which was beyond the control of the researcher.

## Conclusion

The present findings revealed that implementing dynamic educational methods, which involves human cooperation, could improve self-care and health literacy. They can also result in the selection of reliable information and healthy behaviors. Thus, using this educational model by the educational groups can contribute to the promotion of health literacy in practice. This model is suggested as a sociological approach to the health system to improve self-care behaviors and health indices.
